# Treatment and characterization of phosphorus from synthetic wastewater using aluminum plate electrodes in the electrocoagulation process

**DOI:** 10.1186/s13065-019-0628-1

**Published:** 2019-08-14

**Authors:** Dessie Tibebe, Yezbie Kassa, Ashok N. Bhaskarwar

**Affiliations:** 1 Department of Chemistry, College of Natural and Computational Sciences, University of Gondar, P. O. box 196, Gondar, Ethiopia; 2 Department of Biology, College of Natural and Computational Sciences, University of Gondar, P. O. box 196, Gondar, Ethiopia; 3 Department of Chemical Engineering, Institute of Indian Technology Delhi, P.O. Box 110016, New Delhi, India

**Keywords:** Wastewater treatment, Electrocoagulation, Characterization, Aluminum electrode

## Abstract

The main objective of this study is treatment and characterization of phosphorus from synthetic wastewater using aluminum electrodes in the electrocoagulation process. EC experimental setups were designed and different parameters were optimized. The maximum amounts of phosphorus removal efficiencies were observed at pH 7. The phosphorus removal efficiency increases from 85.16 to 97.65% as the temperature increases from 15 to 45 °C, beyond this temperature, there is small effect on removal efficiency. Pollutant removal efficiency increases with an increase in the electrolysis time. At lower initial concentrations the removal efficiencies reached to their maximum values while at the highest initial concentration, the phosphorus removal efficiency was decreased. The increase of current density improves the efficiency of phosphorus removal. Energy and aluminum consumption decreases with increasing initial concentration of phosphorus. Field Emission Scanning Electron Microscope (FESEM) image analysis demonstrated very fine structures for aluminum hydroxide/oxyhydroxides and aluminum phosphate. The existence of the different elemental composition in the sludge was proved by the help of Energy Dispersive X-ray Analysis (EDXS), indicating that the aluminum, oxygen and phosphorus were present in the product. From X-ray diffraction (XRD), Fourier-transform infrared spectroscopy (FT-IR) and Raman analyses of the sludge product, it is concluded that the chemical speciation of the by-products can be mostly aluminum hydroxide and aluminum phosphate.
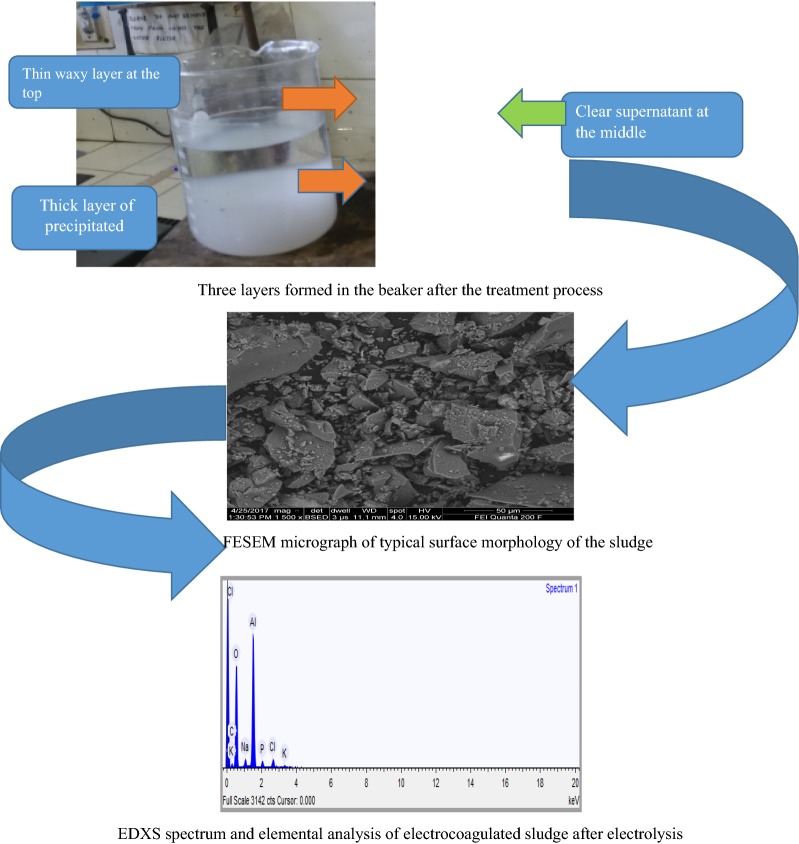

## Introduction

One of the main problems in the twenty-first century is the provision of adequate treated water free from pollutants. At the beginning of 2000, one-sixth of the global population was without access to a clean water supply, leaving over 1 billion people in Asia and Africa alone with a polluted water system [1]. There are various technologies used for the removal of pollutants from wastewater in particular to phosphorus. These technologies are mainly divided into physical, chemical and biological methods. Physical methods are usually too expensive, as in the case electrodialysis or reverse osmosis [2]. In a biological treatment plant, it is necessary to transfer phosphorus from liquid to sludge phase, removal efficiency usually doesn´t exceed 30%, which means that the remaining phosphorus should be removed by other technologies. Therefore, the treatment is not enough to assure complete pretreatment and refining technologies should be added to the treatment process with other advanced technologies which are not economically feasible. Because of the high capital and expensive costs of these techniques, there is a need to use more efficient and cheap methods which requires minimum chemical and energy consumptions [3]. Now a day, electrocoagulation (EC) method gives great attention in wastewater treatment. This technology has been successfully used to remove different kinds of pollutants like phosphorus from wastewater [4]. A significant contribution to the understanding of the removal of phosphorus using coagulation was given by [5]. They suggested that the Al–OH–Al and the Al–PO_4_–Al linkages tend to integrate. Thus, the precipitation is governed by the integrated particles giving the formation of aluminium-hydroxyl-phosphate complexes, Al(OH)_3-x_(PO_4_)_x_, rather than the individual AlPO_4_ and Al(OH)_3_ species. These complexes either adsorb onto positively charged aluminium hydrolysis species or act as further centers of precipitation or nucleation points for aluminum hydrolysis products [5, 6]. Furthermore, this technology is a promising technique for phosphorus removal from wastewater because it is simple, selective, effective, ability in multi-pollutant removal and economical, result in less sludge production and therefore experience minimal disposal problems [7, 8].

There have been different studies on various aspects of the phosphate removal from wastewater using electrocoagulation process [2, 6, 9, 10]. However, studies on comparison between the different anode and cathode Al electrode systems in both for the removal of phosphorus from wastewater and the characterization of the electrodes before and after treatments as well as the sludge formed after the treatment using FESEM, EDXS, XRD, FTIR and Raman spectroscopy are very limited. Therefore, the main objective of this study is the treatment and characterization of phosphorus from synthetic wastewater using aluminum plate electrodes in the electrocoagulation process.

## Materials and methods

### Experimental setup

**Fig. 1:**
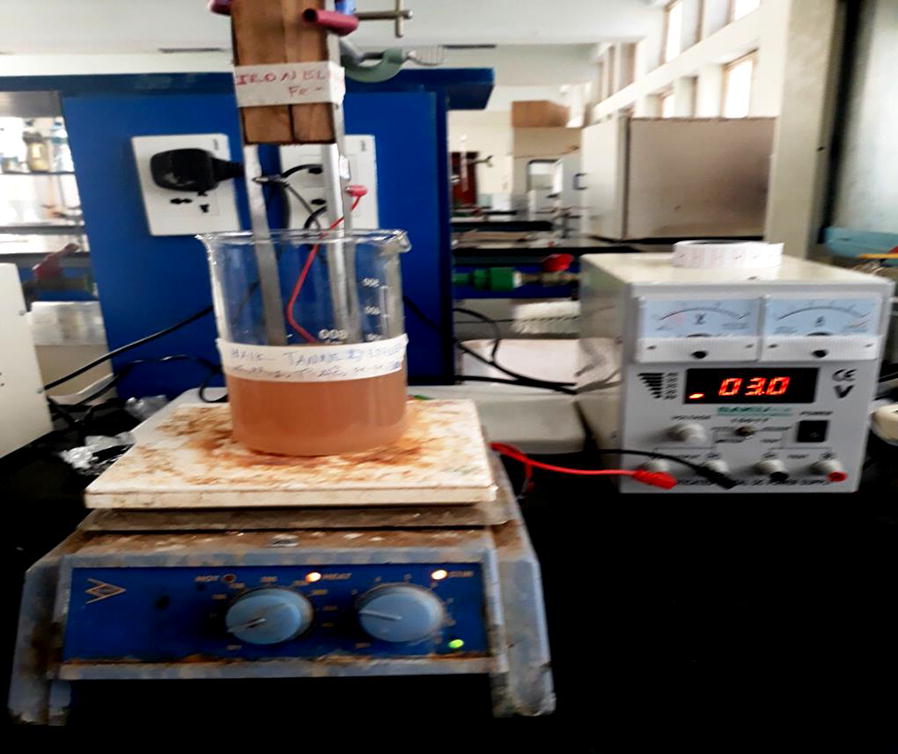
Experimental setup for the electrocoagulation process using aluminum electrode systems

The experimental setups for the designed EC process were explained as follows (Fig. 1): For each run a 0.9 L of synthetic wastewater was mixed with 0.1 g of sodium chloride which was used as increasing electrical conductivities of the solution. The solutions were placed into the 1 L beaker. NaOH and HCl solutions were used to adjust the pH. In separate different electrode systems with the same dimension of Aluminum electrode were used in EC technique. External power supply was applied through the different electrode systems using a DC power supply. A 10 mL sample solution was taken at different time intervals in each run. The location of the drawn samples was kept constant for each run. The submerged portion of an electrode was 10 × 3 × 1 cm though its actual dimension was 20 × 3 × 1.5 cm. The distance between the electrodes was kept constant at 2 cm and the effective submerged area was 30 cm^2^.

During the EC process the synthetic wastewater in the beaker was mixed continuously with a 30 mm magnetic stirrer at 200 rpm. During the experiments, temperature and pH of the synthetic wastewater was measured by a pH meter (pHTestr 30).

### Calculation of removal efficiency

The removal efficiencies for Phosphorus were calculated as follows:

1 $$Removal\;efficiency\,\left( \% \right) = \left( {\frac{{C_{o} - C_{f} }}{{C_{o} }}} \right)*100$$

where; C_o_ is the initial phosphorus concentration (mg/L) and C_f_ is the final phosphate concentration (mg/L) [9].

### Chemicals and reagents

All chemicals used were analytically graded and used without further treatment. The chemicals used in the present work were sodium hydroxide (reagent grade, ≥ 98%), concentrated hydrochloric acid, concentrated sulfuric acid (reagent grade, 95–98%), potassium antimony tartrate, ammonium molybdate (ACS reagent, ≥ 99%), ascorbic acid (ACS reagent, ≥ 99%), potassium di-hydrogen phosphate (ACS reagent, ≥ 99%), and sodium chloride (AR grade, ≥ 99%). All the working solutions were prepared using distilled water except the cleaning of electrode with 5% HCl solution.

### Apparatus and equipment

The apparatus and equipments used in the experiments were UV–Visible Spectrophotometer (Perkin Elmer Lambda 25, USDA); portable pH meter (pHTestr 30, China);DC Power Supply Regulator (L3210 Regulated DC Power Supply 0–16 V/0–2 A, Aplab Limited, India), Al plate electrodes; Magnetic Stirrer (30 mm) with Hot Plate (Remi 5 MLH plus, India), Digital mass balance, Oven Dry (Macro Scientific Works Pvt Ltd, India), Field Emission Scanning Electron Microscope (FESEM) (FEI Quanta 200 F SEM, Netherland), EDXS (Oxford xmax 80 mm^2^, Netherland), XRD, FT-IR Spectrophotometer (Nicolet 6700 FT-IR spectrometer, Thermo Scientific, India) and Raman Spectrometer (Micro Raman Spectrometer, UK).

### Measurements of phosphorus concentration from synthetic wastewater

The analysis of phosphorus was measured colorimetrically using ascorbic acid method following the standard procedures outlined in [11]. The filtered sample was mixed with ammonium molybdate that forms molybdo-phosphoric acid with any phosphate present in the water sample. The acid is then reduced by ascorbic acid to a blue complex known as molybdenum blue. The color intensity, which is proportional to the concentration of phosphate in the water sample, was then measured by a UV Visible spectrophotometer at a wave length of 880 nm. Then, concentration of phosphorus was calculated from standard calibration curve.

## Results and discussion

### General observation on the electrocoagulation process

**Fig. 2:**
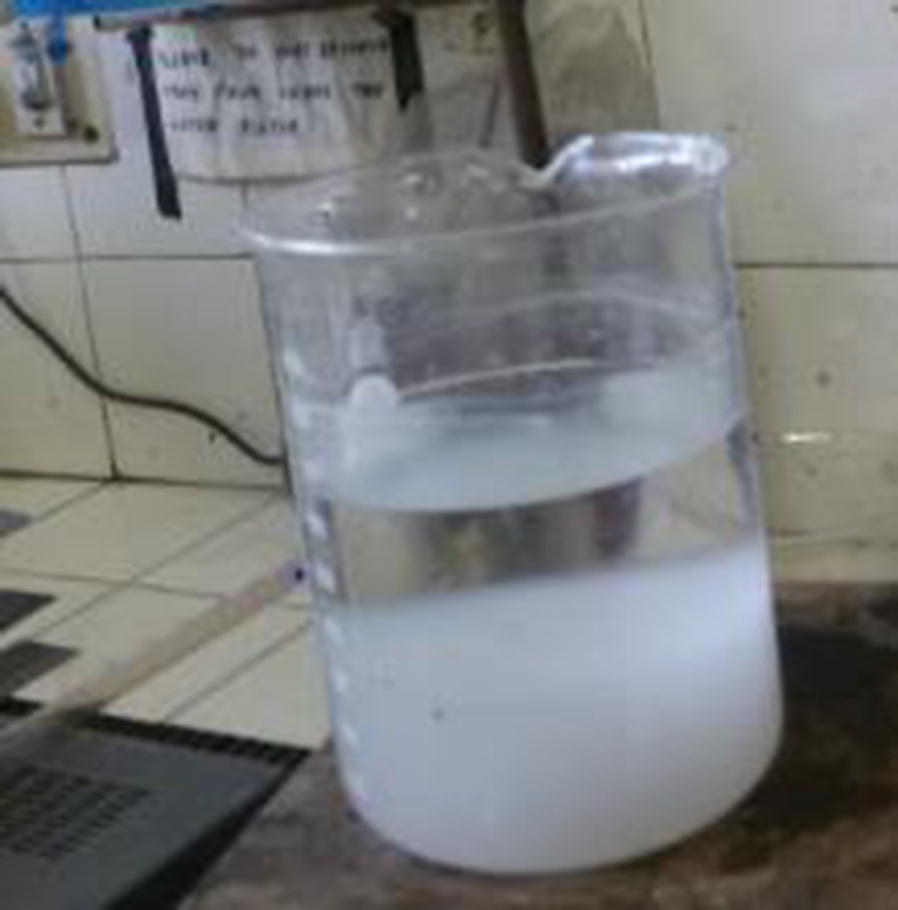
Three layers formed in the beaker after the electrocoagulation process

Generally, there is an electro-deposition mechanism in electrocoagulation process due to electrochemical reactions [12]. At the positive side (anode), it can be seen that there were some aluminum oxide deposited on the surface of electrode due to dissolution of anode, generation of Al(OH)_3_ for coagulation and the pH of the solution around this area was acidic media. Meanwhile, negative side (cathode), aluminum plate electrode surface was cracked because the release of too much hydrogen bubbles which help flocculated particles to float out of the aqueous solution and there were also basic media around this electrode. Moreover, there were three layers formed in the beaker after the electrocoagulation process (Fig. 2). A very thin waxy layer of flocs formed at surface, clear supernatant at the middle and a thick layer of precipitated sludge at the bottom. Aqueous solution in electrocoagulation cell that used aluminum plates as electrodes become white, right after electrocoagulation process.

### Evaluation of phosphorus removal efficiencies using different Al electrode connection systems in electrocoagulation process

For removal efficiencies of phosphorus using Al electrodes in different electrolytic systems in different electrolysis time with constant current density were explained in Table 1. From the table removal efficiency of the anode–cathode–anode and pit anode–cathode–anode electrolytic cells were higher than that of anode–cathode, pit anode–cathode and cathode–anode–cathode electrolytic cells in all the electrolysis time and different concentrations. This is attributed to a significant increase in the removal rate with increasing aluminum anode plate and pitting the anode electrode. As the electrolysis time increased, the surface of the anode plate was passivated due to the presence of an oxide film. An additional anode plate delayed the passivation, thus improved the coagulation effect and increase removal efficiencies of phosphorus from synthetic wastewater [13].

**Table 1:** Comparison between the different Al electrode systems in the removal efficiency of phosphorus from synthetic wastewater

Time (min)	Initial conc. (mg/L)	A–C	A–C pit	C–A–C	A–C–A	A–C–A pit
5	25	32.24 ± 1.32	53.48 ± 1.53	61.85 ± 2.00	71.96 ± 1.37	86.03 ± 0.60
15		63.22 ± 1.30	54.22 ± 0.44	77.82 ± 0.03	60.22 ± 1.66	88.29 ± 0.41
30		67.45 ± 2.13	74.45 ± 0.59	78.19 ± 2.05	86.43 ± 0.53	89.86 ± 0.18
50		74.56 ± 1.43	88.06 ± 1.26	93.01 ± 0.36	94.22 ± 0.79	94.06 ± 0.97
80		82.15 ± 0.68	92.15 ± 0.68	92.76 ± 0.60	94.00 ± 0.71	94.94 ± 1.19
120		88.81 ± 0.94	96.31 ± 0.61	94.48 ± 0.66	95.47 ± 0.18	97.48 ± 0.55
160		29.36 ± 0.62	94.33 ± 0.71	96.33 ± 0.17	93.27 ± 0.33	95.42 ± 0.27
5	50	34.12 ± 0.66	44.36 ± 2.27	64.47 ± 4.18	65.64 ± 0.72	58.35 ± 0.53
15		55.83 ± 1.29	54.12 ± 0.66	70.05 ± 0.21	75.03 ± 1.79	82.54 ± 0.43
30		58.80 ± 1.53	73.83 ± 0.54	78.10 ± 1.02	80.47 ± 0.31	84.63 ± 1.03
50		76.42 ± 1.41	76.30 ± 1.07	80.18 ± 1.23	91.72 ± 0.61	90.45 ± 0.79
80		88.81 ± 0.94	88.92 ± 1.23	93.77 ± 0.54	95.05 ± 1.15	96.86 ± 0.21
120		93.35 ± 1.05	94.12 ± 0.19	96.00 ± 0.06	96.54 ± 0.48	98.03 ± 0.41
160		93.35 ± 1.05	93.35 ± 1.05	95.68 ± 0.63	94.27 ± 0.62	95.56 ± 0.41
5	75.00	45.81 ± 0.67	55.81 ± 0.67	62.35 ± 11.61	49.99 ± 8.53	43.58 ± 0.73
15		46.33 ± 1.89	68.08 ± 3.11	72.33 ± 1.4	66.69 ± 1.09	58.31 ± 0.50
30		55.48 ± 0.75	85.48 ± 0.75	75.31 ± 0.92	84.44 ± 8.10	63.98 ± 1.23
50		57.34 ± 0.97	86.34 ± 0.05	87.90 ± 4.28	88.06 ± 8.22	77.57 ± 0.37
80		74.98 ± 1.47	87.23 ± 1.00	91.98 ± 1.03	89.31 ± 8.21	92.11 ± 0.83
120		92.70 ± 0.46	92.70 ± 0.46	94.84 ± 1.05	89.01 ± 8.35	94.28 ± 0.58
160		90.69 ± 0.59	90.44 ± 1.16	93.16 ± 1.01	85.74 ± 8.06	92.89 ± 0.95
5	100	28.68 ± 2.79	60.18 ± 2.10	56.45 ± 1.43	66.38 ± 1.47	40.05 ± 2.37
15		47.00 ± 0.20	62.00 ± 2.69	58.81 ± 1.52	69.28 ± 0.41	55.66 ± 0.47
30		53.56 ± 3.87	71.06 ± 1.04	73.87 ± 1.65	80.83 ± 1.00	62.79 ± 0.23
50		56.23 ± 0.55	80.98 ± 0.13	89.16 ± 0.97	89.83 ± 0.27	76.93 ± 0.11
80		68.39 ± 0.41	88.39 ± 0.41	88.70 ± 1.02	95.35 ± 0.07	87.46 ± 1.04
120		90.80 ± 1.24	94.19 ± 0.50	90.82 ± 0.55	97.55 ± 0.03	94.86 ± 1.20
160		88.44 ± 0.79	90.80 ± 1.24	87.05 ± 1.16	93.82 ± 0.19	92.63 ± 0.84

A–C–: anode–cathode; C–A–C–: cathode–anode–cathode; A–C–A–: anode–cathode–anode electrode system

From the result, as the electrolysis times were above 120 min, the average removal efficiency reached more than 90% and 75% in anode–cathode–anode system and anode–cathode system, respectively, indicating that the removal rates significantly increased with increasing the electrolysis time in the concentration of phosphorus below 100 mg/L. These results showed that the removal rates increased with the electrolysis time. Therefore, in the ranges of the test factors, the order of this electrolytic cell on the removal rate was depends on the electrolysis time and the number and nature of Al electrode systems [14].

The results of the anode–cathode–anode and pit anode–cathode–anode systems in this experiment are similar to two anode and two cathode electrode systems as reported by [9, 15]. Therefore, this result has an advantageous to minimize aluminum electrode consumption.

## Optimization of the different parameters in electrocoagulation process for phosphorus removal from synthetic wastewater

### Effect of initial phosphorus concentration on the removal efficiency

**Fig. 3:**
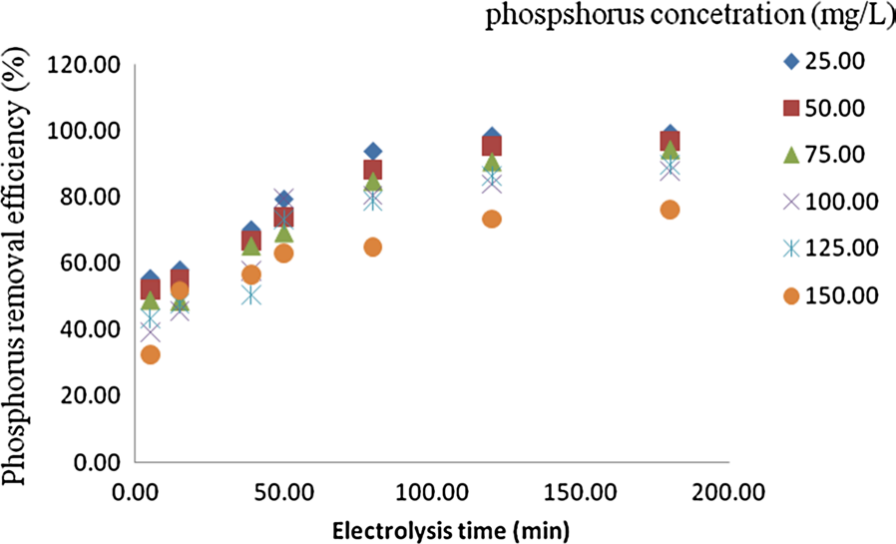
Effect of initial concentration on the percentage of removal of phosphorus in anode–cathode system (pH = 7, current density (J) = 10 mA/cm^2^, NaCl = 0.9 g/L, temperature = 25 °C and time = 120 min)

**Fig. 4:**
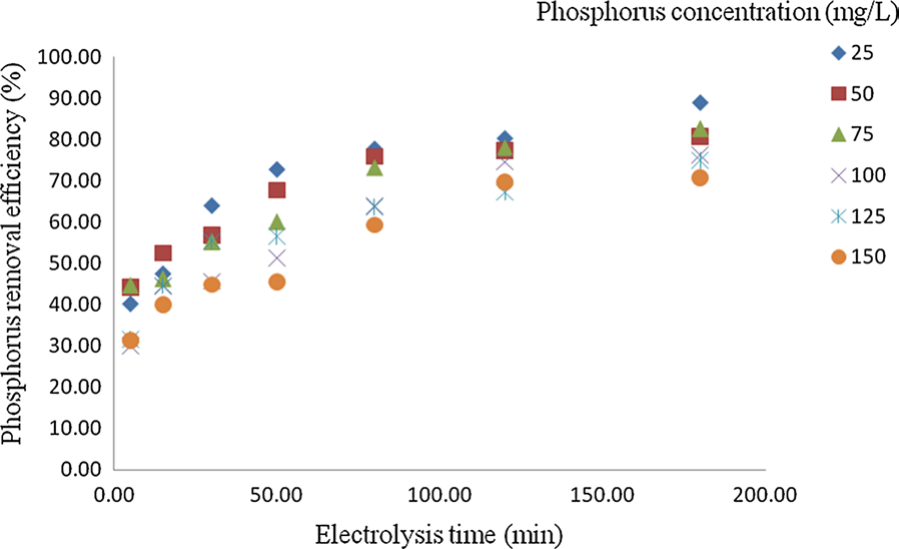
Effect of initial concentration on the percentage of removal of phosphorus in anode–cathode–anode system (pH = 7, current density (J) = 10.0 mA/cm^2^, NaCl = 0.9 g/L, temperature = 25 °C and time = 120 min)

The phosphorus removal efficiency is influenced by the different initial phosphorus concentrations ranged from 25 to 150 mg/L (Figs. 3, 4). It can be observed that increase in concentration at constant electrolysis time and current density result in decrease in removal efficiencies in maximum values from 88.97 to 70% and 98.50 to 76% at 120 min in the anode–cathode and anode–cathode–anode electrolytic systems, respectively. This is due to the fact that the number of metal hydroxide flocs formed may be insufficient to sediment the greater number of phosphate ions at higher initial phosphorus concentration. Similarly [7, 9, 13, 16, 17] were studied on the removal of phosphate from wastewater using electrocoagulation techniques. Their result revealed that the increase in phosphorus concentrations leads to decrease in removal efficiency.

Moreover, the following main reactions takes place in the EC reactor, the OH^−^produced in the cathode is immediately react with the aluminum ions oxidized from the anode enter in the solution and produce aluminum hydroxides. Subsequently, this process initiates polymerization reactions when aluminum hydroxide particles reached a sufficient concentration and react with phosphate ions present in the solution and formed aluminum phosphate (Eqs. 2, 3) and sediment in the solution and helps to reduce phosphorus from the wastewater [3, 16, 17]. These results are also consistent with the findings in [18] studied on the removal of phosphates by electrocoagulation using aluminum electrodes and investigated the removal efficiency with several initial concentrations of phosphates, ranging from 10 to 200 mg/L in a series of six pair of aluminum electrodes. They found that a decrease in the removal efficiency from 100 to 88.2% with increasing the initial concentration of phosphates.

Furthermore, a study on phosphate removal reaction mechanism by aluminum electrode is available on the literature by [3], which involves phosphate interaction with aluminum as shown in Eqs. (4) and (5):

2 $${\text{Al}}\left( {\text{aq}} \right) + 3 {\text{H}}_{ 2} {\text{O}}{\rightarrow}{\text{Al}}\left( {\text{OH}} \right)_{ 3} + 3 {\text{H}}^{ + }$$

3 $${\text{Al}}\left( {\text{aq}} \right) + {\text{PO}}_{ 4}^{ 3- }{\rightarrow} {\text{AlPO}}_{ 4}$$

### Effects of current density on the phosphorus removal efficiency

**Fig. 5:**
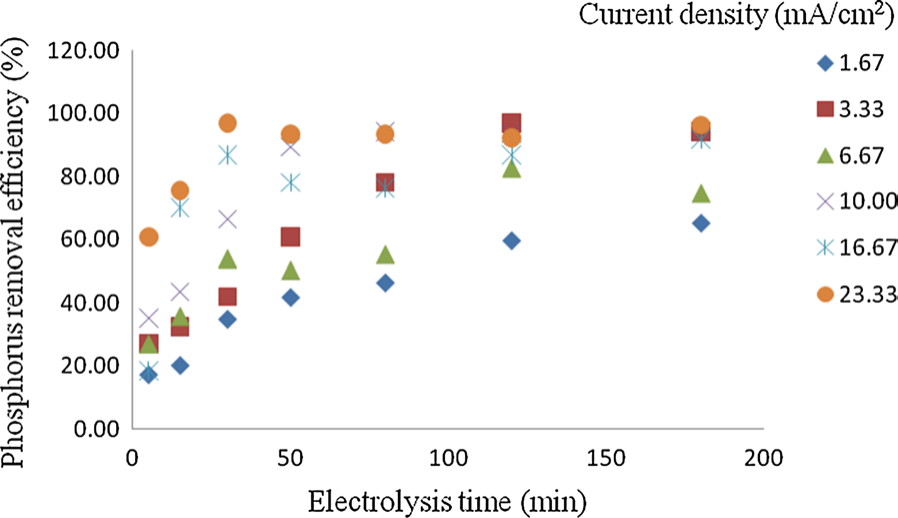
Effect of current density on the percentage of removal of phosphate (C_o_ = 50 mg/L, NaCl = 0.90 g/L, pH = 7, temperature = 25 °C and time = 120 min)

Batch electrocoagulation experiments were conducted for different current densities within a different electrolysis time. Figure 5 shows the effect of changing the electrolysis time on phosphorous removal efficiency. It was found that the phosphorous removal efficiency increases with increasing the time of electrolysis until reaches equilibrium at 120 min and constant as the electrolysis time increases. This phenomenon was caused by the limited adsorption capacity of the aluminum hydroxide flocs at higher electrolysis time. Figure 5 also shows that an increase in current density from 1.67 to 23.33 mA/cm^2^ increases the removal efficiency of phosphorus from 61 to 98%. This is ascribed to the fact that at higher current densities the dissolution of anode to Al^3+^ ions increases according to Faraday’s law. Al^3+^ ions undergo hydrolysis and the resulting aluminum hydroxides produce more sludge with a consequent significant removal of phosphorus due to phosphorus adsorption on Al(OH)_3_ and its polymeric compounds [13, 19]. At low pH, phosphate ions can be removed by precipitation through the formation of AlPO_4_ insoluble (4) [20].

4 $${\text{Al}}^{3 + } + {\text{PO}}_{4}^{3 + } \to {\text{AlPO}}_{4}$$

Furthermore, more hydrogen bubbles are generated at the cathode; these bubbles improve the degree of mixing of aluminum hydroxides and phosphorus and enhance the flotation ability of the cell with a consequent increase in the removal efficiency. Besides, it was demonstrated that bubbles density increases and their size decreases with increasing current density, resulting in a greater upwards flux and a faster removal of phosphorus and sludge flotation [13, 19].

### Effect of electrocoagulation time

**Fig. 6:**
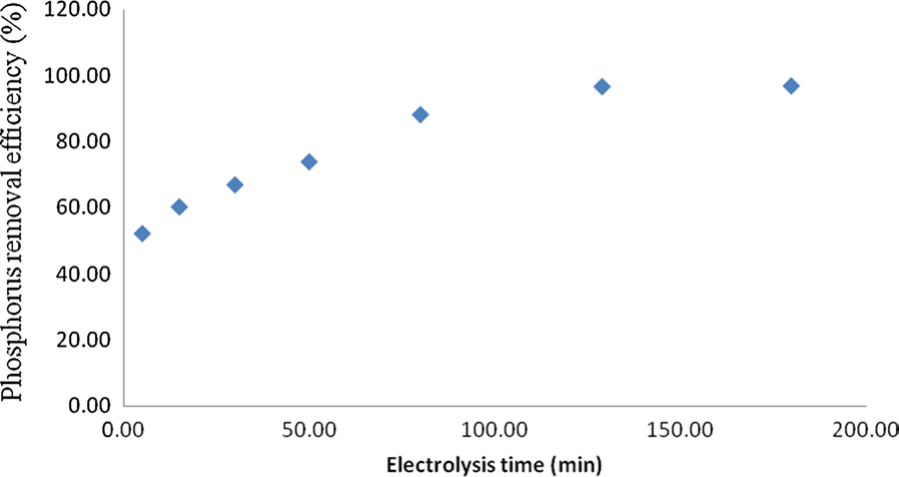
Effect of electrocoagulation time on the percentage of removal of Phosphorus (Co = 50 mg/L, current density = 10 mA/cm^2^, NaCl = 0.9 g/L, pH = 7, temperature, 25 °C)

Figure 6 showed the effect of electrocoagulation time towards percentage removal of phosphorus. The figure revealed that the maximum phosphorus removal efficiency was 97% at 120 min electrocoagulation time. These percentage removal values were induced by highest potential of electrocoagulation process. Thus, these percentage removal values are selected as optimum values of the process. Percentage removal of phosphorus would remain the same values even the process increase its electrocoagulation time longer than 120 min. Time of electrocoagulation is a time provided to the process to generate metal hydroxides and to complete coagulation of phosphorus. Highest removal efficiency has been achieved due to the increase amount of aluminum hydroxide coagulant produced parallel with increment in electrolysis time [21].

For an electrolysis time beyond the optimum electrolysis time of 120 min, the phosphorus removal efficiency does not increase as sufficient numbers of flocs are available for the removal of the phosphorus. Similar results were reported by [13]. Besides, increment in treatment time contributed to higher rate of bubble generation, which helped to remove phosphorus and flocs of lower density and size by gas floatation. The rate of bubbles generation also increased and the size of bubble decreased which induced a higher removal of phosphorus by hydrogen gas flotation [2].

### Effect of pH on the removal efficiency of phosphorus

**Fig. 7:**
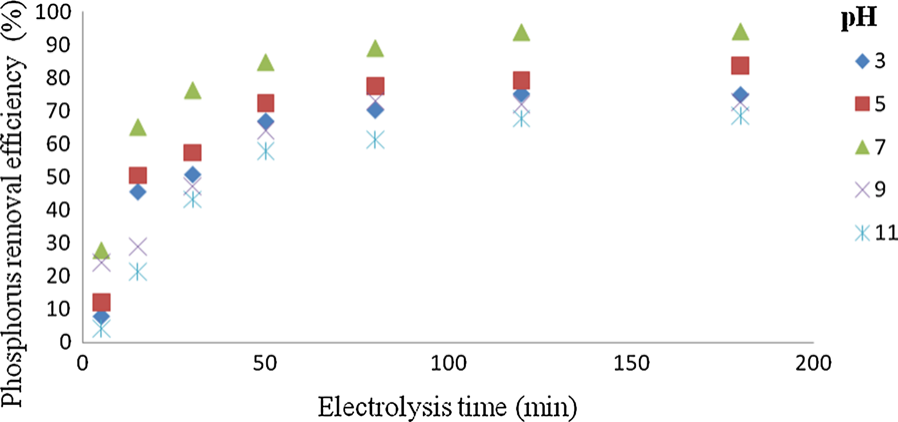
Effect of the pH value of the solution on the percentage of phosphorus (C_o_ = 50 mg/L, current density = 10 mA/cm^2^, NaCl = 0.9 g/L, temperature = 25 °C and time = 120 min)

The removal of phosphorus is completely depends on the initial pH value and at the lowest and highest initial pH values, phosphorus removal efficiencies were very low (Fig. 7). The maximum and minimum removal efficiencies of 94% and 68% at the electrolysis time of 120 min were obtained at pH 7 and pH 11, respectively. The effect of pH on the process performance was explained as follows: At lower pH, the oxide surfaces showed a net positive charge and adsorption of an ionic phosphate was enhanced by columbic attraction [2]. At higher pH, the oxide surface had a net negative charge which can repulse the anionic phosphate in the solution [2].

**Fig. 8:**
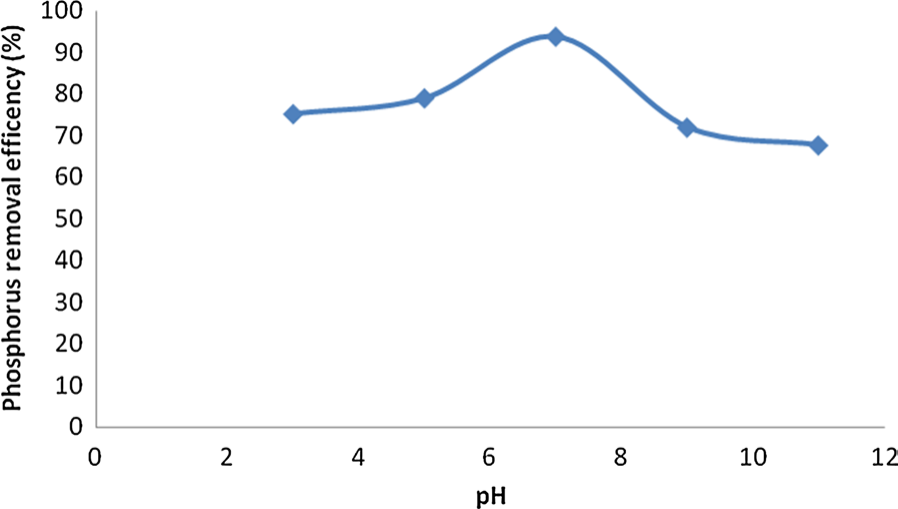
Effect of pH on percentage of removal (C_o_ = 50 mg/L, time = 120 min, current density (J) = 10 mA/cm^2^, NaCl = 0.9 g/L, temperature = 25 °C)

Figure 8 showed the effect of pH after EC treatment would increase for acidic influent but decrease for alkaline influent. Many investigators demonstrated that the removal efficiency of phosphorus decreases at more acidic and alkaline pH solution. This is attributed to amphoteric behavior of Al(OH)_3_ which leads to soluble Al^3+^ cations in acidic media and to monomeric anions Al(OH)_4_^−^ in alkaline media. It is well known that both species are not useful for water treatment. If pH of the solution is neutral, all the aluminum produced at the anode formed polymeric species (Al_13_O_4_(OH)_24_^7+^) and precipitated Al(OH)_3_ leading to more removal efficiency [18, 22, 23]. In this study, the maximum amounts of phosphorus removal efficiencies were observed at pH 7. In addition, there is also O_2_ evolution reaction leading to pH decrease.

### Effect of temperature

**Fig. 9:**
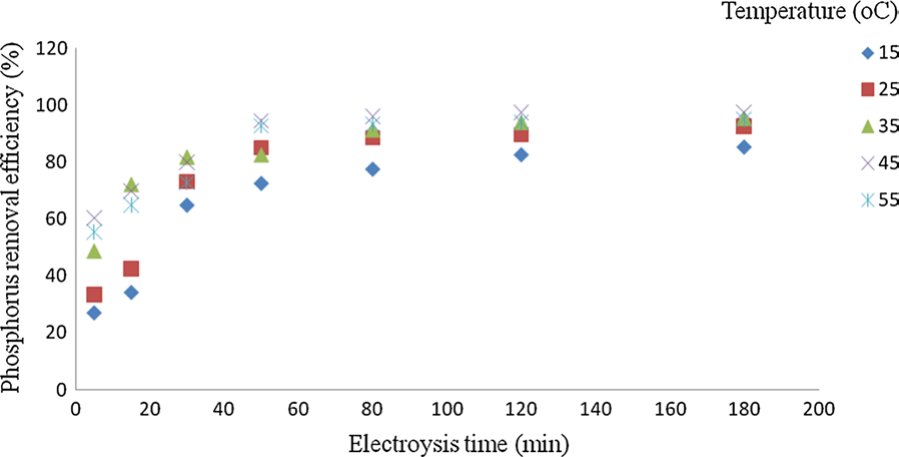
Effect of temperature on the phosphorus removal efficiency (C_0_: 50 mg/L, current density: 10 mA/cm^2^, NaCl = 0.9 g/L, pH = 7)

Figure 9 depicts the effect of solution temperature on the electrocoagulation reactor performance. The result showed that removal of phosphorus increases by increasing temperature up to 45 °C. The phosphorus removal efficiency increases from 85.16 to 97.65% as the temperature increases from 15 to 45 °C, beyond this temperature, there is small effect on removal efficiency. If temperature increases, both rate of Al^3+^ hydrolysis to Al(OH)_3_ and the diffusivity of Al^3+^ increase according to the Stokes–Einstein’s equation with a consequent increase in the rate of mass transfer of Al^3+^ from the anode surface to the solution bulk. The phosphorus removal efficiency decreases above 45 °C, this is explained by the fact that higher solution temperatures increase both anode and cathode electrode passivation by the formation of protective aluminum oxide layers, which reduces Al^+3^ ions dissolution and consequently the electrocoagulation reactor performance [10, 24].

## Kinetic models for the analysis of the electrocoagulation process

**Fig. 10:**
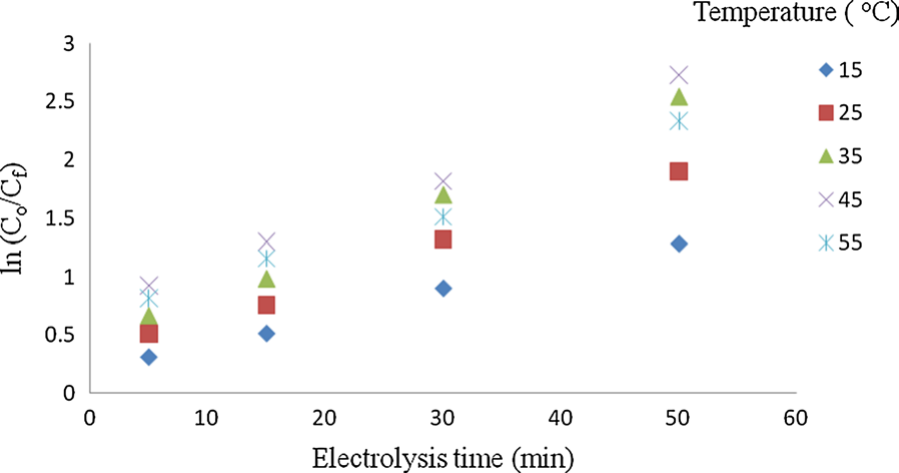
ln (C_o_/C_f_) vs. time at different solution temperature

The kinetics of phosphorus removal by electrocoagulation has been successfully described by the first order rate equation as explained in Eqs. 2.1 and 2.2 [15]. Figure 10 shows that the electrocoagulation reaction kinetic data obtained in this study is well fitted by the first order rate equation given above and the calculated mass transfer coefficient increases with temperature within the reaction temperature range reported.

**Fig. 11:**
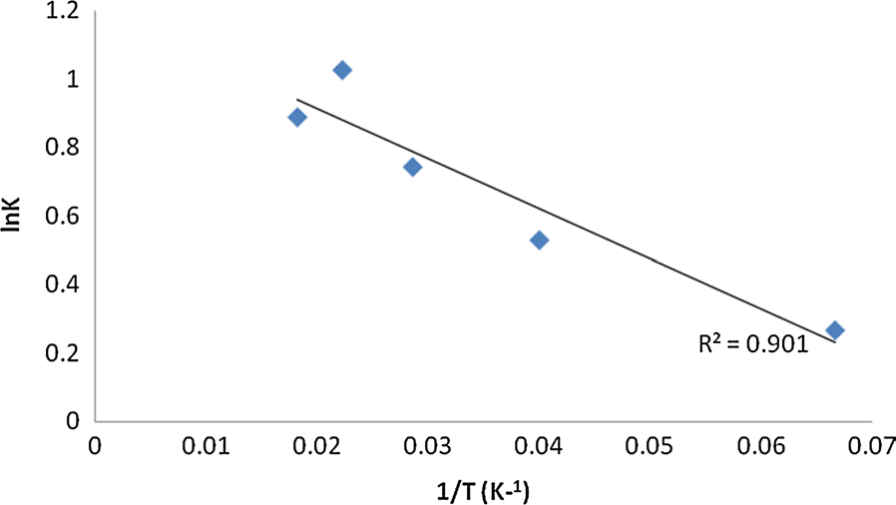
ln K vs. 1/T

Figure 11 shows that the temperature effect on the rate of electrocoagulation process is well described by Arrhenius equation. Moreover, data of Fig. 11 shows that the activation energy of the process was about 142 J/mol, which can confirm that the reaction is diffusion controlled and an increase in solution flow rate through the reactor would indeed improve the electrocoagulation reactor performance [15, 24].

**Fig. 12:**
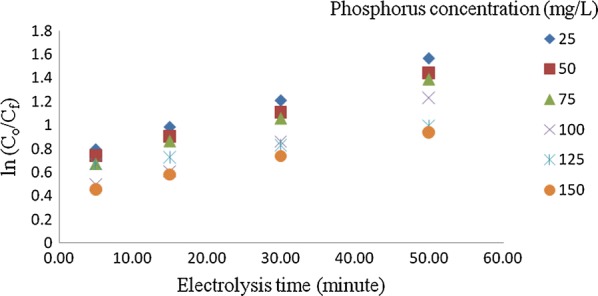
Effect of initial phosphorus concentration on the kinetics of the reaction

Besides, the effect of initial phosphorus concentrations on the kinetic study was also an important issue in order to examine the order of the reaction. Figure 12 showed the effect of initial concentration of phosphorus on the kinetics of the reaction. Finding showed that the phosphorus removal by electrochemical process is a first order reaction because the semi-log plot of means of residual phosphorous versus reaction time is a straight line [25].

Furthermore, the present data fit (Eq. 2.2), however, the slightly two slopes after few minutes are distinguished indicating the presence of two rates of removal. In first stage which occurs during few minutes, the rate is higher than the rate in second stage (Fig. 12). The fast rate of removal in the first few minutes is attributed to the flocs are still free so the adsorption rate is very fast. The low rate of removal in second stage is due to the fact that the flocs becomes exhausted (the number of free flocs is few) so the adsorption rate is very slow. Table 2 shows the calculated rate constants of the first order reaction). This result is agreed with [7].

**Table 2:** Rate constants, K and R^2^ values for the first order

Phosphorus concentration, C_o_/mg/L	K	R^2^
25	0.0267	0.998
50	0.0256	0.998
75	0.0199	0.998
100	0.0153	0.993
125	0.0193	0.994
150	0.0124	0.997

## Energy and aluminum electrode consumptions vs. electrolysis time at different current density

**Fig. 13:**
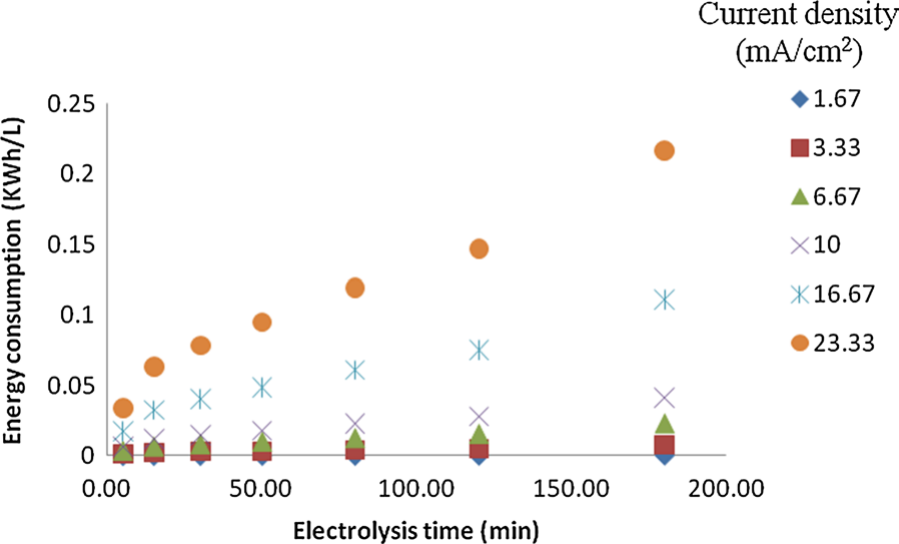
Variation of energy consumption vs. electrolysis time at different current density

As seen in Fig. 13, with increase of current density, the energy consumption was improved sharply for 1.67 mA/cm^2^, the energy consumption was 0.0003 kWh/L at 120 min, but for 23.33 mA/cm^2^ the energy consumption was 0.141 kWh/L at 120 min. With increase of current density, the needed current and potential were improved, as a result, the energy consumption increase accordingly. From the results obtained in Fig. 15, it could be concluded that while the current density was 3.33 mA/cm^2^ and the electrolysis time was 120 min, about 97.05% of phosphorus could be removed from the synthetic wastewater, while the current density was 23.33 mA/cm^2^ and the electrolysis time was 180 min, 96.54% of phosphorus could be removed from the synthetic wastewater.

**Fig. 14:**
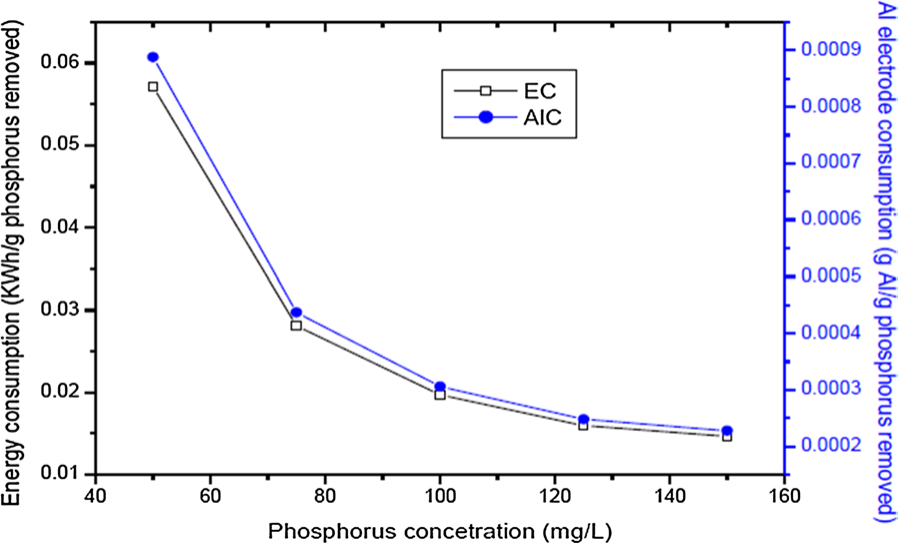
Effect of different phosphorus concentration on energy consumption and Al consumption (current density = 10 mA/cm^2^, temperature = 25 °C, pH = 7)

In order to assist in assessing the economic feasibility of electrocoagulation in comparison with other techniques, the energy consumption and Al metal consumption were also calculated according to Eqs. (2.4 and 2.5) [26]. Based on the models, the variation of electrical energy consumption and electrode consumption with current density and phosphorus concentration were also presented in Fig. 14.

As seen from Fig. 14, the experimental energy consumption values ranged from 0.31 to 2.24 kWh/g phosphorus removed 1.96 to 1.27 kWh/g phosphorus removed and from 5.47 to 0.44 kWh/g phosphorus removed. The variation of aluminum consumption ranged from 0.004 to 0.017 g aluminum/g phosphorus removed for initial phosphorus concentration, 0.016 to 0.011 g aluminum/g phosphorus removed for initial phosphorus concentration and from 0.035 to 0.0041 g aluminum/g phosphorus removed for initial phosphorus concentration. It is clear from these figures that energy consumption increases with increasing the current density and decreases with increasing concentration of phosphorus removed. Aluminum consumption increases with increasing the current density, and decreases with increasing concentration of phosphorus removed [9, 13, 26, 27].

## Characterization of sludge and aluminum plate electrodes using microscopic and spectroscopic methods for the removal of phosphorus from synthetic wastewater

### Characterization of electrocoagulated sludge and aluminum plate electrodes using FESEM and EDXS analyses

**Fig. 15:**
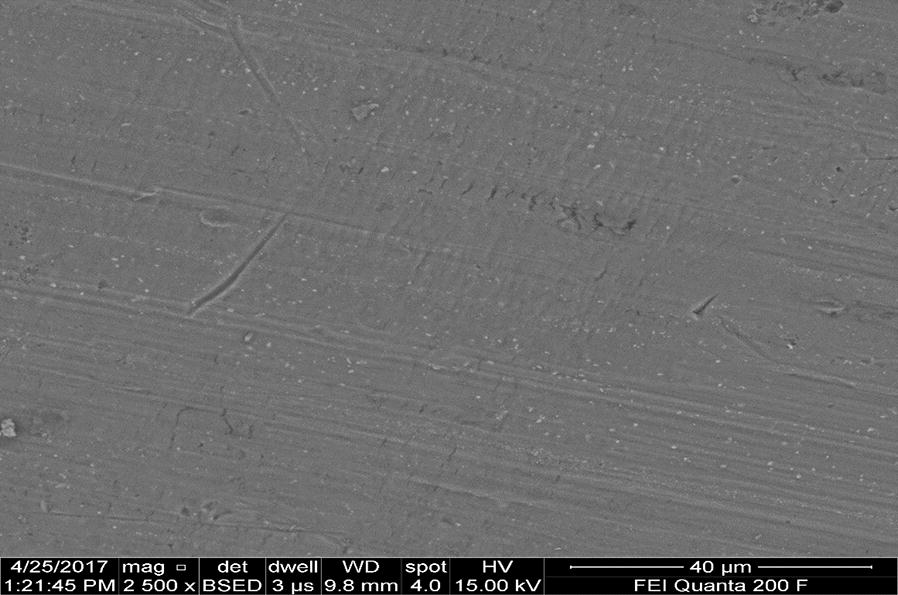
FESEM micrography of typical surface morphology of aluminum plate electrode before treatment

Figure 15 showed the FESEM microscopic images of the Al plate electrode before treatment. The microscopic image indicates uniform and smooth but there are some scratching and impurities on the electrode surface during electrode preparation.

Figures 17 and 19 provide good evidence that both anodic and cathodic dissolution of aluminum at the anode and cathode electrodes as shown by FESEM image taken after electrolysis, respectively. According to the Pourbaix diagram, aluminum is passive in the pH range of 4–8.5 [28]. Beyond this range, aluminum corrosion occurs in aqueous solutions since its oxide is soluble either in acidic and alkaline media, yielding to Al^3+^ ions in the former and [Al(OH)_4_]^−^ ions in the latter. During electrolysis, the significant increase of the local pH at the cathode vicinity due to hydrogen evolution induces “chemical” attack of aluminum and its hydroxide film as shown in (Eqs. 5, 6). According to several authors [10, 15, 22, 28] chemical attack of both anode (pH < 4) and cathode (pH > 10) can occur due to the acidity and alkalinity produced at their vicinity, respectively:

At the anode vicinity:

5 $$2{\text{Al}} + 6{\text{H}}^{ + } \to 2{\text{Al}}^{3 + } + 3{\text{H}}_{2}$$

At the cathode vicinity:

6 $$2{\text{Al}} + 6{\text{H}}_{ 2} {\text{O}} + 2 {\text{OH}}^{ - } \to 2[{\text{Al(OH)}}_{ 4} ]^{ - } + 3{\text{H}}_{2}$$

7 $${\text{Al(OH)}}_{ 3} + {\text{OH}}^{ - } \to [{\text{Al(OH)}}_{ 4} ]^{ - } .$$

This implies that as OH^−^ ion concentration rises at the cathode vicinity, uniform thinning of the aluminum electrode overwhelms the pitting corrosion by Cl^−^ ions attack. This observation is in agreement with findings of some investigations dealing with corrosion of aluminum in alkaline media [7, 22, 29]. Furthermore, as the current density decreases the amount of aluminum generated increases (Fig. 17). Since electrolyses were carried out at constant charge loading, the duration time of electrolysis is as long as current density is low. Consequently, the contact time of the cathode with the local alkalinity produced at its surface during H_2_ evolution is longer. This gives rise to the chemically generated aluminum.

**Fig. 16:**
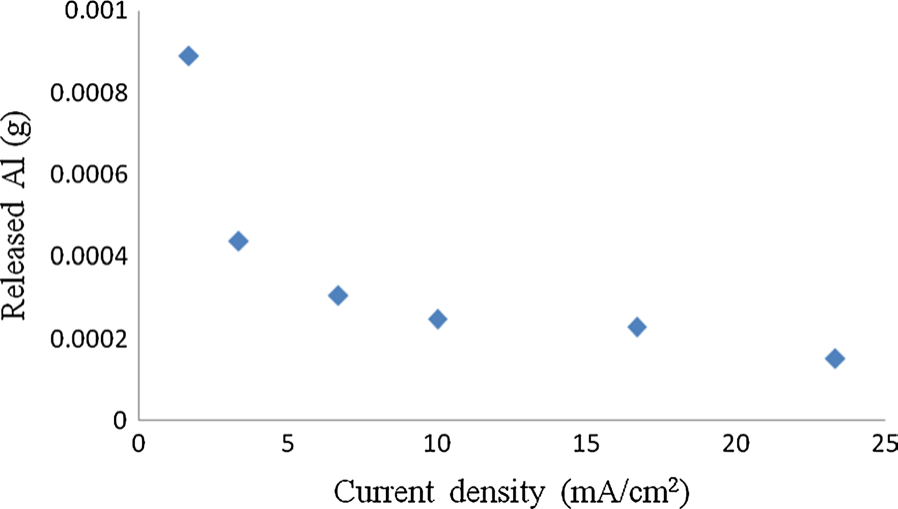
Variation of the amount of released aluminum at the electrodes as a function of current density

It is interesting to point out that the shape of the curve, showing the amount of dissolved aluminum at the electrodes (Fig. 16), is similar to other literatures [22, 29].

**Fig. 17:**
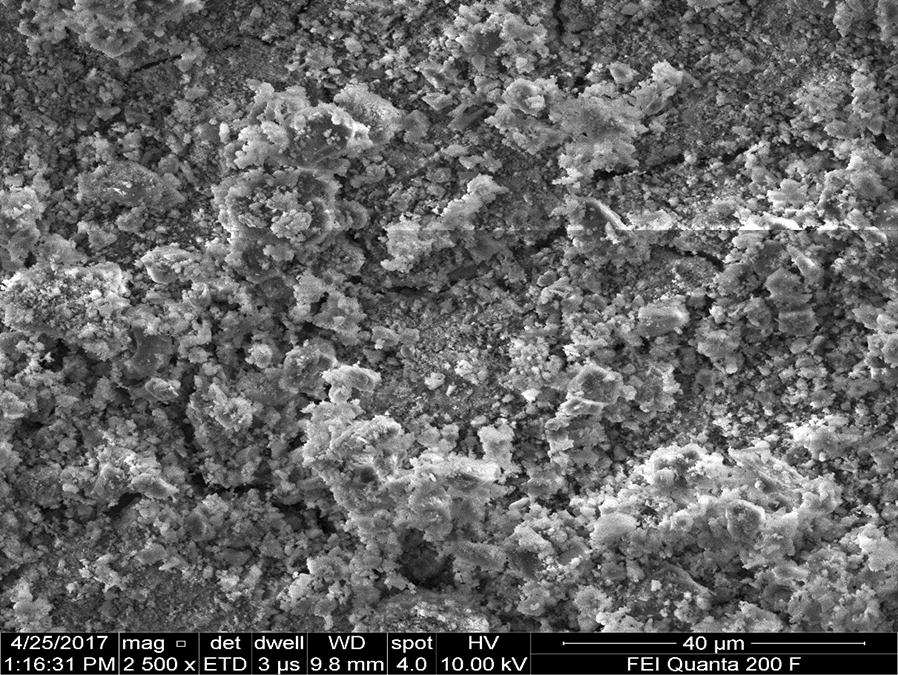
Typical FESEM micrography of a typical pit on anodic aluminum surface after electrolysis

The surface morphology of the aluminum anode electrode in the submerged regions changed in structure after electrolysis (Fig. 17) indicating the interaction of the dissolution of the Al electrode and adsorption of phosphorus on the electrode surface.

**Fig. 18:**
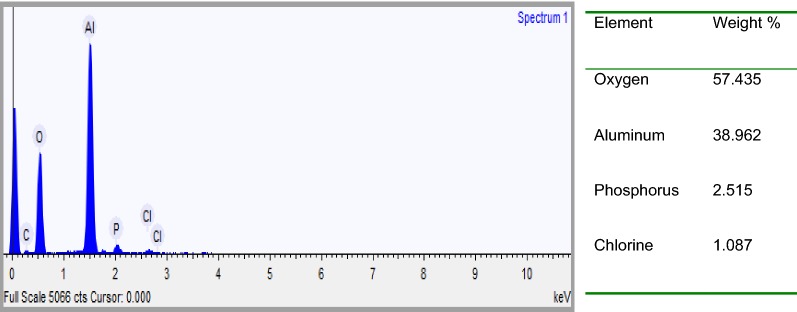
EDXS spectrum and elemental analysis of anodic aluminum surface after electrolysis

**Fig. 19:**
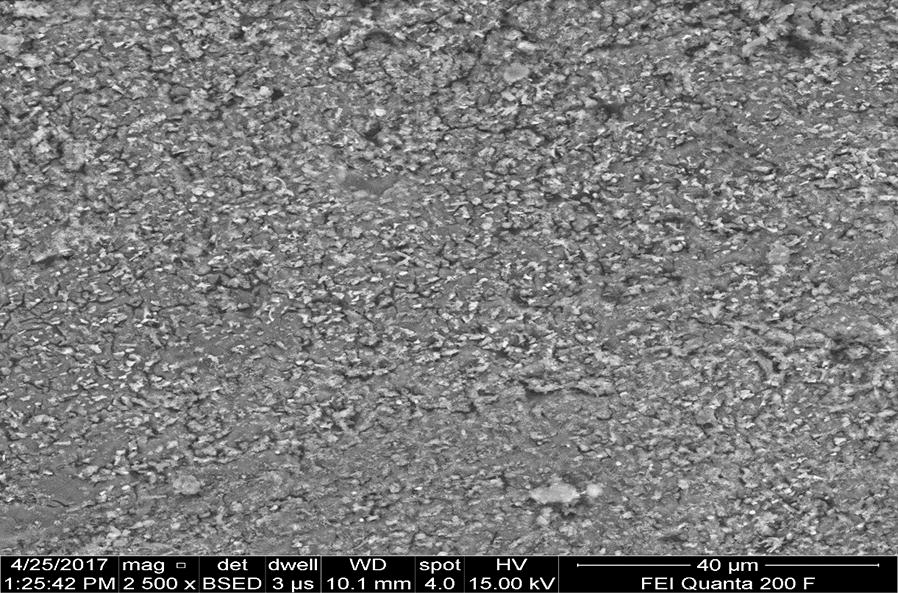
FESEM micrography of typical surface morphology of aluminum cathode electrode after electrolysis

EDXS was used to analyze the elemental constituents of anode and cathode aluminum electrodes (Figs. 17, 18). As seen in Figs. 19 and 21, the elemental analysis by EDXS showed elements in the following order as per mass %: O > Al > P > Cl and Al > O > P in the anode and cathode electrodes, respectively. High contents of oxygen (57.44%), aluminum (38.96%), phosphorus (2.52%) and chlorine (1.09%) in the anode and aluminum (66.77%), oxygen (32.38%) and phosphorus (0.852%) in the cathode indicate a high aluminium oxide and hydroxide formation as well as aluminum phosphate at both electrodes during EC process. The analysis also showed high content of aluminium in both electrodes due to the use of aluminium electrode in the treatment. The peak of chlorine was found in the anode due to oxidation of chlorine on the anode electrode (Eqs. 9, 10). Chlorine was introduced during pH adjustment by using HCl and the added NaCl electrolyte and the following are the main reactions which might be probably the deposition of aluminum hydroxide chlorine on the anode (Eqs. 9, 10) [30].

Anodic reactions:

8 $$H_{2} O \to OH^{ - }$$

9 $$Cl^{ - } \to Cl^{ \cdot } + e^{ - }$$

10 $$2Cl^{ \cdot } \to Cl_{2}$$

11 $$Al\left( {OH} \right)_{2}^{ + } + Cl^{ - } \to Al \left( {OH} \right)_{2} Cl$$

**Fig. 20:**
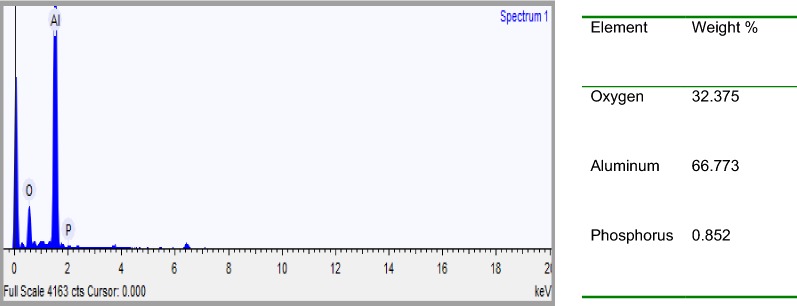
EDXS spectrum and elemental analysis of aluminum cathode electrode after electrolysis

Furthermore, it can be seen that there are some aluminum oxide and aluminum phosphate deposited on the surface of the anode electrode due to dissolution of anode, generation of aluminum ions (aluminum hydroxide) for coagulation and deposition of the precipitate on the electrode (Figs. 19, 20). Meanwhile, in the cathode electrode, the aluminum electrode surface was cracked because the release of too much hydrogen bubbles which help flocculated particles to float out of the aqueous solution (Fig. 20). This result is supported by [31].

Regarding the precipitated sludge analysis, there were three layers formed after the electrocoagulation process in the reactor. A very thin waxy layer of flocs formed at surface, clear supernatant at the middle and a thick layer of precipitated sludge at the bottom of the beaker due to gravity (Fig. 2) [31]. At the end of the process, treated water became clear and isolated. Theoretically, the sludge contained phosphate and polymeric aluminium which act as a coagulant in the process. The percentage of aluminum hydroxide or aluminium oxide in sludge is normally greater than phosphate. According to [29], in aluminum electrode used, the high charged poly-nuclear hydroxyl aluminium complexes, such as Al_2_(OH)_2_^4+^ Al_7_(OH)_17_^4+^, Al_13_(OH)_34_^5+^, Al_3_(OH)_4_^5+^, Al(OH)_6_^3−^, Al(OH)_7_^4−^ and AlO_2_^−^, were produced.

The sludge was easily settled down at the bottom of the beaker because of density of aluminium is denser than aqueous solution. Therefore, the sludge generated by electrocoagulation was analyzed for particle shape and elemental composition. The solid sludge samples were characterized by field emission scanning electron microscope (FESEM) coupled with an energy dispersion X-ray spectroscopy (EDXS).

**Fig. 21:**
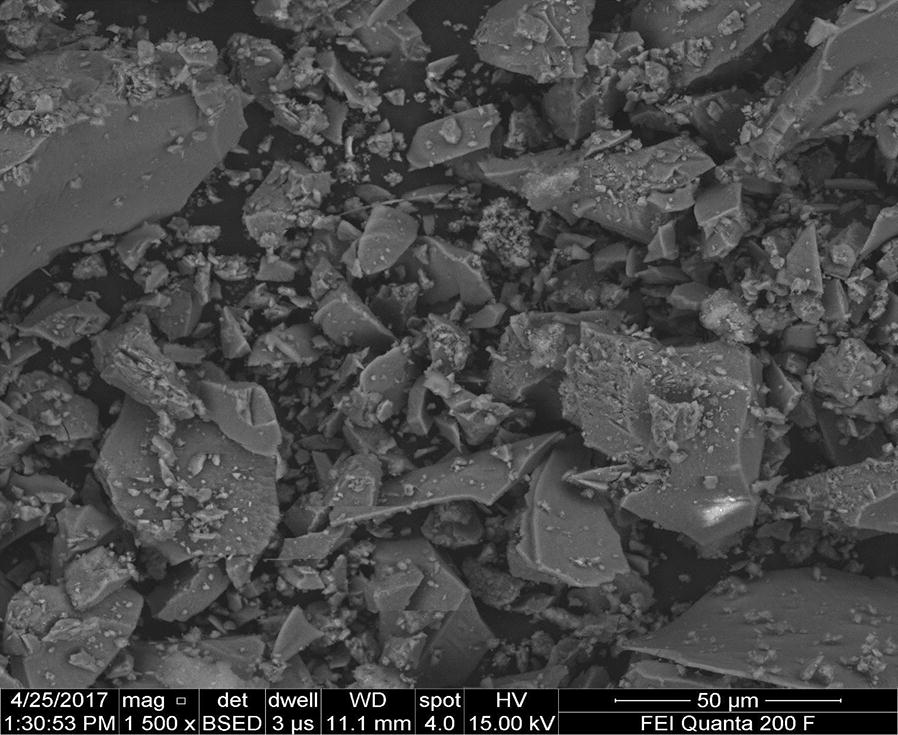
FESEM micrograph of typical surface morphology of the sludge

FESEM is used to evaluate the structural features of the sludge generated by the electrochemical process. As shown in Fig. 21, the FESEM image indicates the presence on the surface of different crystalline structures and sizes with different aggregations were deposited at the bottom of the reactor during electrocoagulation at micrometer size.

**Fig. 22:**
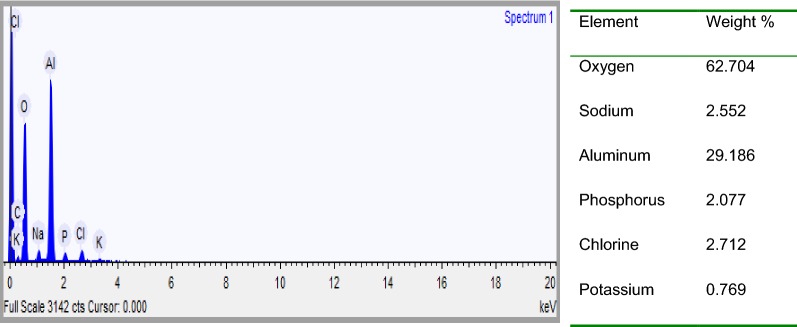
EDXS spectrum and elemental analysis of electrocoagulated sludge after electrolysis

EDXS was used to analyze the elemental constituents of sludge (Fig. 22). The presence of Al, O, P, Na, K and Cl in the spectrum provides direct evidence that the presence of the expected products like aluminum hydroxide and aluminum phosphate in the sludge. Other elements detected in the sludge come from the pH adjustment, conducting electrolyte, chemicals used in the experiments and the scrap impurities of the anode and cathode [23]. As seen in Fig. 24 the elemental analysis by EDS showed elements in the following order as per mass %: O > Al > Cl > Na > P > K. High contents of oxygen (62.70%) and aluminum (29.19%) indicate a high oxides and hydroxides of aluminum content in the electrocoagulated sludge due to the formation of coagulants for precipitate formation with pollutants during EC process.

The analysis also showed high content of aluminium (6.8%) due to the use of aluminium electrode in the treatment. The elemental analysis of sludge mainly revealed presence of phosphorous, which was the main pollutant to be removed from the synthetic wastewater. As seen in Fig. 24 in EDXS analysis, the expected products of Al(OH)_3_ and AlPO_4_ are available in the medium. The sludge produced by the process of phosphate removal using electrocoagulation method is usually a mixture of Al(OH)_3_ and AlPO_4_ although the AlPO_4_ precipitation is favored over Al(OH)_3_ [15, 32].

### XRD characterization

**Fig. 23:**
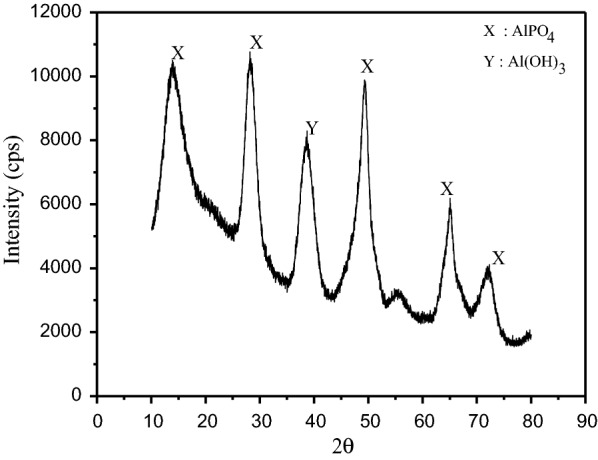
XRD diffractograms of the sludge obtained after electrolysis

In order to determine the species in the precipitate, XRD diffractograms has also been used with the 2θ scans recorded from 10° to 80°. As seen in Fig. 23, Al(OH) _3_ and AlPO_4_ are available in the medium. The sludge produced by the process of phosphorus removal using electrocoagulation method is usually mainly a mixture of Al(OH)_3_ and AlPO_4_ although the AlPO_4_ sludge is favored over Al(OH)_3_. Extremely narrow lines indicate a very well-ordered crystalline structure in the structure of tetragonal in shape of aluminum phosphate. The XRD result is supported by [15, 19, 23, 33–36].

### FTIR analysis

**Fig. 24:**
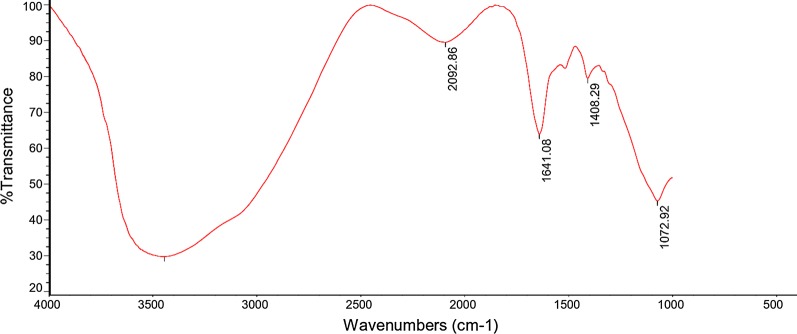
FTIR spectrum of the resulting sludge using aluminum plate electrodes

FTIR spectra of the sludge obtained after electrocoagulation process is shown in Fig. 24. The typical feature of the FTIR spectrum of the sludge varies depending on the functional group of the obtained compound. The large wide peak at 3450.75 cm^−1^ is due to the O–H stretching vibration in the Al(OH)_3_ structures [23]. The wide and smooth peak that is observed at 2092.86 cm^−1^ corresponds to O–H bond belonging to the hydrogen phosphate group. The peaks at 1641.08 and 1408.29 cm^−1^ indicate bent vibration of H–O–H and Al–H stretching, respectively. The result is agreed with [23]. Moreover, the band observed around 1072.92 cm^−1^ is due to symmetric stretching mode of PO_4_^3−^ in the crystalline structure of AlPO_4_ (Fig. 24). This result is supported by [19, 34–36].

Besides, the dominant sludge color was white crystalline when electrolysis time was short and blue-black when electrolysis time was long. After drying the sludge the color of precipitate was a mixture of blue-green and white aluminum phosphate and white hydroxyl aluminum phosphate. The hydroxyl aluminum phosphate has good adsorption and coagulation, and can help to improve the rate of phosphorus removal from sewage. Therefore, longer electrolysis time lead to better effect of coagulation on phosphates. This result is supported by [14, 16, 17, 19] studies on removal of phosphate from drinking water by electrocoagulation process, Phosphorus Removal from wastewater in Johkasou Sewage treatment tank by electro-coagulation and phosphate removal efficiency electrocoagulation wastewater using iron and aluminum electrodes, respectively.

### Raman spectra analysis

**Fig. 25:**
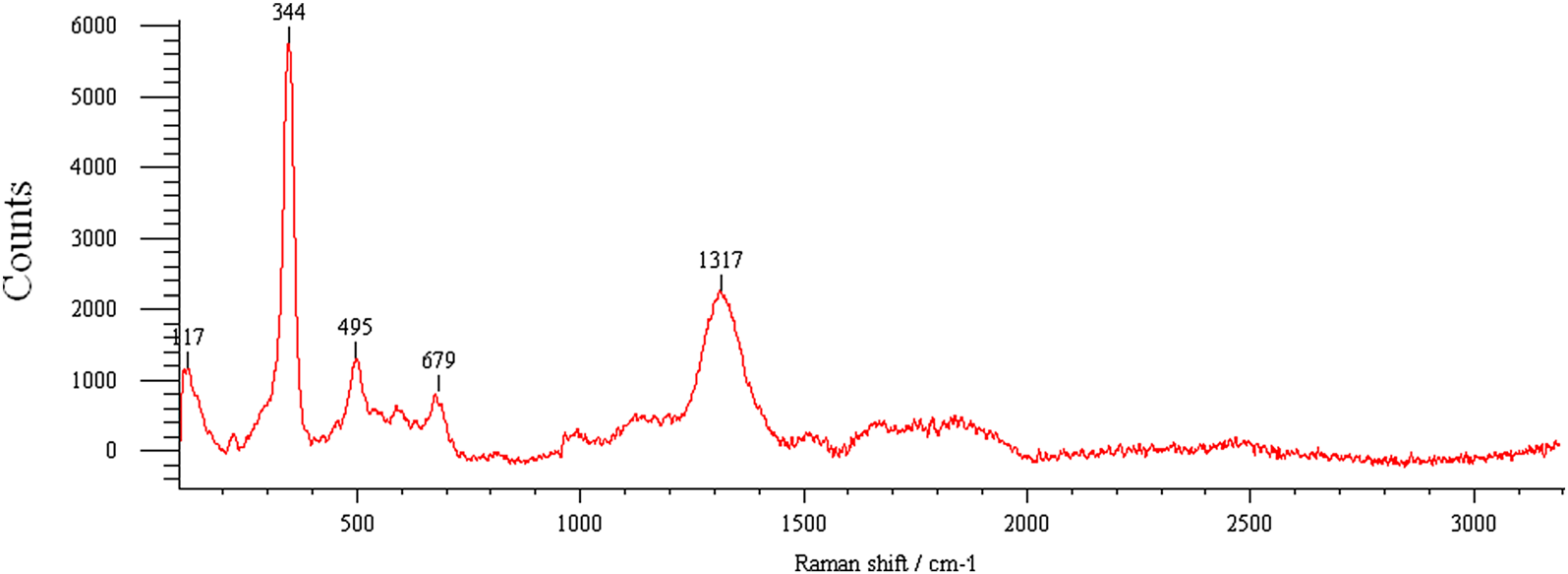
Raman spectra for the sludge obtained after electrolysis of phosphorus from synthetic wastewater using aluminum plate electrodes

To investigate the functional groups of the sludge product in the removal of phosphorus from synthetic wastewater Raman spectroscopy analysis was also carried after electrocoagulation process. The Raman spectrum shown in Fig. 25 display a number of absorption peaks, which indicated the presence of different types of functional groups in the sludge. The peak at 1317 cm^−1^ is due to the P=O symmetric stretch. Peak at 679 cm^−1^ has been attributed to motions of bridging oxygen in P–O–P chains, originates from the P–O–P symmetric stretch. Besides the symmetric stretching vibration, the other most intense bands belong to the O–P–O bending mode. The intense Raman band at 497 cm^−1^ is ascribed to this Vibrational mode (Fig. 25). The sharp peak at 344 cm^−1^ has been assigned to bending vibrations of phosphate (PO_4_^3−^). This result is also in agreed with [37–39] studies on the Raman and infrared spectroscopic analysis of the phosphate mineral wardite NaAl_3_(PO_4_)_2_(OH)_4_2(H_2_O), Vibrational spectroscopic of the phosphate mineral churchite (REE)(PO_4_)_2_H_2_O and Vibrational studies of strontium antimony phosphate glass, respectively.

## Conclusion

In this study different parameters were optimized based on the performance of phosphorus removal efficiency. The obtained results were very interesting and promising technology for wastewater treatment in the real world. The characterization of FESEM analysis showed the different morphologies of the Al plate electrodes before and after electrolysis as well as the sludge product. Moreover, results of EDXS were proved the presence of aluminum, oxygen and phosphorus in the product. From the results of microscopic and spectroscopic techniques, it is concluded that the chemical speciation of the by-products were mostly aluminum hydroxide and aluminum phosphate.

## Data Availability

The datasets used and/or analyzed during the current study are available from the corresponding author.
